# Early Mammogram Screening's Impact on Early Breast Cancer Detection in Underserved Populations

**DOI:** 10.7759/cureus.48616

**Published:** 2023-11-10

**Authors:** Oluwaremilekun Tolu-Akinnawo, Kikelola Oyeleye, Taiwo Talabi, Oghanim I Ogwu, Akintomiwa Akintunde, Bukola Olagbende, Oshuare Polly, Su Yi, Rosalena Muckle

**Affiliations:** 1 Internal Medicine, Meharry Medical College, Nashville, USA; 2 Internal Medicine, Meharry medical college, Nashville, USA

**Keywords:** early identification and diagnosis, primary care physician, health surveillance, screening mammogram, tennessee, breast cancer research

## Abstract

Background

Breast cancer remains a pressing public health challenge in the United States, ranking as one of the most prevalent cancers and the second leading cause of cancer-related deaths among women. This study investigates the effectiveness of early mammogram screening in underserved populations.

Methods

Data from female patients receiving primary care at a tertiary hospital in Nashville between January 2022 and January 2023 were retrospectively analyzed. Inclusion criteria encompassed females aged 40 or older with initial mammogram screenings before turning 50. Exclusions included genetically or environmentally related risk factors, cosmetic motivations, age above 50 at first screening, and screenings prompted by physical exams.

Results

Of 150 eligible women aged 40-49, the majority (n=121, 80.7%) had normal findings, 18.0% (n=27) had benign lesions, and 1.3% (n=2) had suspicious/malignant lesions. About 30.7% (n=46) underwent additional testing due to suspicious masses, with ultrasounds and diagnostic mammograms being common. The breast malignancy positivity rate was 1.33% (n=2) for the study population and 4.3% among those requiring additional testing. The positivity rate for the population of Black American descent is 1% (n=2), and for the Hispanic population, it is 6.25% (n=1).

Discussion

Breast cancer remains a significant concern, with disparities in screening guidelines and varying age of diagnosis. Overdiagnosis and false positives are challenges, with our study highlighting potential benefits in early screening, particularly for populations with unique risk factors, such as smokers. However, the study's limitations, including a small sample size and demographic bias, necessitate larger, more diverse studies to establish stronger correlations. Shared decision-making in early mammogram screening is emphasized.

Conclusion

Early mammogram screening in the 40-49 age group may detect breast cancer cases, but guidelines remain inconsistent. The study recommends early screening at age 40, with awareness of potential advantages and disadvantages. Larger, more comprehensive studies are needed to inform breast cancer screening practices better.

## Introduction

Breast cancer remains a significant challenge in public health within the United States, maintaining its status as one of the most frequently diagnosed malignancies and the leading cause of cancer-related mortality among women [1]. The alarming statistics reflect the gravity of this issue, with an estimated 2.26 million (95% UI, 2.24-2.79 million) new cases of breast cancer diagnosed in 2020 alone worldwide [2, 3]. Astonishingly, one in eight women (13%) in the United States will receive a breast cancer diagnosis during their lifetime, and this incidence continues to rise [4]. Projections for 2023 indicate an estimated 297,790 new cases in females and 2,800 cases in males [4]. It is worth noting that breast cancer also contributes to 684,996 deaths (95% UI, 675,493-694,633) worldwide, making it the leading cause of cancer death in women [2, 3]. Numerous factors have been linked to an elevated risk of breast cancer, including sex, age, family history, race, geographic location, and socioeconomic status. Female sex represents a major risk factor partly due to enhanced hormonal stimulation, particularly estrogen and progesterone [5]. Age similarly reigns supreme as one of the most significant risk determinants among these factors, yet prevailing clinical guidelines exhibit inconsistencies [6, 7]. However, current literature reports that about 80% of breast cancer diagnoses are in individuals above 50 years, and at the same time, more than 40% are above 65 years old [8-10]. Moreover, the burden of breast cancer demonstrates regional disparities, with a notable divide between rural and urban areas. In the context of our study location, Tennessee, breast cancer held the highest incidence rate among all cancers from 2015 to 2019. It ranked as this region's second leading cause of cancer-related mortality [11]. In 2023, breast cancer remains a significant health challenge, with an estimated 6,210 cases recorded in this area (Tennessee), trailing only behind lung and prostate cancer [11].

The current study unfolds within the backdrop of an academic-affiliated community hospital in Nashville, Tennessee. Its overarching goal is twofold: to enhance the provision of cost-effective, evidence-based guidelines for mammogram screening, drawing upon contemporary research findings, socioeconomic factors, and existing literature. Additionally, this endeavor assumes the role of a quality improvement project, striving to elevate our institution's patient-centered care standard.

## Materials and methods

This study aims to investigate the efficacy of early mammogram screening in facilitating the early detection of breast cancer within underserved populations.

Methodology

We conducted a comprehensive retrospective analysis of clinical data involving female patients who were actively receiving primary care at a tertiary hospital during the timeframe spanning from January 2022 to January 2023. This retrospective analysis was undertaken with the exempt approval of the Institutional Review Board (IRB) at Meharry Medical College under the assigned approval number FWA00003675. Given the nature of our study, which required an extensive search through the hospital's electronic medical records to extract pertinent information on eligible candidates, it was imperative to secure IRB clearance. Utilizing the hospital's electronic medical records, our search process was meticulously tailored to adhere to predefined inclusion and exclusion criteria. These criteria were established to ensure the precise identification of relevant cases for our study. To facilitate the systematic collation of data, we employed a division of group members, each assigned with specific responsibilities to gather the necessary information. The amassed data was then meticulously organized within an Excel spreadsheet (Microsoft, Redmond, Washington), ensuring the proper categorization and classification of each patient's details. Following the initial data compilation, the Excel spreadsheet underwent a rigorous review process conducted by two independent group members. This meticulous review process was instituted to ascertain the eligibility of each candidate and to eliminate any potential duplicates or inaccuracies that could have inadvertently surfaced during the data collection phase. The culmination of these efforts led to the subsequent analysis of the study, as outlined and detailed comprehensively in the dedicated results section of this research article. As part of our methodological framework, we meticulously defined the inclusion and exclusion criteria to ensure the accuracy and relevance of our study's patient cohort.

The inclusion criteria were deliberately set to encompass female patients aged 40 years and older who had undergone their initial mammogram screening before reaching the age of 50. On the contrary, the exclusion criteria were designed to account for specific circumstances where the presence of genetically or environmentally related risk factors warranted an earlier initiation of breast cancer screening or where mammogram screenings were primarily sought for cosmetic purposes rather than for the detection of breast cancer. Additionally, patients above 50 years of age at the time of their first mammogram screening, as well as those whose screenings were prompted solely by physical examination findings, were also excluded from the study cohort.

To ensure a comprehensive analysis, our study took into account various intervals for mammogram screenings, including both annual and biennial occurrences. Furthermore, we incorporated cases where additional imaging or biopsies were conducted due to suspected breast masses or lesions, providing a more holistic perspective on the diagnostic journey of the patients within our study cohort. The final composition of our study cohort comprised a total of 150 eligible patients, reflecting a diligent adherence to the specified inclusion and exclusion criteria. While our data analysis was based on the available information, it is crucial to acknowledge the inherent limitations associated with our relatively modest sample size, which may impact the generalizability of our findings within a broader context.

Statistical analysis

Categorical variables were presented using frequencies and percentages, while the continuous variable, age, was summarized using means and standard deviations. We also computed the breast cancer positivity rate for the overall sample and among those who underwent additional testing.

## Results

In this study, we analyzed data from 150 women who had undergone at least one screening mammography between the ages of 40 and 49. The participants had a mean age of 44.9 years (standard deviation: 2.9). The racial distribution of the participants ranked as follows: Black (n=100, 66.7%), White (n=26, 17.3%), Hispanic (n=16, 10.7%), and 'other race' (n=8, 5.3%). Most participants (n=121, 80.7%) had no history of benign or malignant masses, showing normal findings. Approximately 18.0% of participants had encountered benign lesions or masses on at least one occasion (n=27), while only 1.3% had suspicious or malignant lesions (n=2) (Table 1). Out of the 150 women who underwent screening mammograms, 30.7% (n=46) required additional testing due to suspicious masses (Table 2). Most of those necessitating further evaluation underwent either an ultrasound and diagnostic mammogram (n=16) or ultrasound alone (n=16) (Table 3). The breast malignancy positivity rate within the study population stood at 1.33% (n=2), while among those who underwent additional testing, it was 4.3%. The two individuals diagnosed with breast cancer (confirmed with biopsy/tissue diagnosis) were a 49-year-old Black woman and a 47-year-old Hispanic woman. The positivity rate for the African population is 1% (n=2), and 6.25% (n=1) in the Hispanic population. Table 4 shows the frequency of mammogram screenings done.

**Table 1:** Frequency of abnormal lesion findings

Type of lesion	Frequency (N)	Percent (%)	Valid percent (%)	Cumulative percent (%)
Benign lesions/ mass	27	18	18	18
Normal	121	80.7	80.7	98.7
Suspicious lesions/ malignant	2	1.3	1.3	100
Total	150	100	100	

**Table 2:** Frequency of additional testing done

Additional testing	Frequency (N)	Percent (%)	Valid percent (%)	Cumulative percent (%)
No	104	69.3	69.3	69.3
Yes	46	30.7	30.7	100
Total	150	100	100	

**Table 3:** Types of additional testing done

Type of additional testing	Frequency (N)	Percent (%)	Valid percent (%)	Cumulative percent (%)
No additional testing	104	69.3	69.3	69.3
Mammogram	7	4.7	4.7	74
Mammogram and biopsy	1	0.7	0.7	74.7
Ultrasound	16	10.7	10.7	85.3
Ultrasound and biopsy	2	1.3	1.3	86.7
Ultrasound and mammogram	16	10.7	10.7	97.3
Ultrasound, mammogram, and biopsy	4	2.7	2.7	100
Total	150	100	100	

**Table 4:** Frequency of mammogram screening done

Frequency of screening	Frequency (N)	Percent (%)	Valid percent (%)	Cumulative percent (%)
Annual	90	60	60	60
Biannual	33	22	22	82
Irregular	18	12	12	94
Once	9	6	6	100
Total	150	100	100	

## Discussion

Despite the introduction of mammogram screening over two decades ago, the incidence of breast cancer diagnoses has risen, while the prevalence of metastatic breast cancer has remained stable. This has raised concerns regarding the uncertain benefits and substantial risks associated with screening average-risk patients. The decision to undergo mammogram screening typically occurs during primary care provider visits. However, inconsistencies in recommendations for average-risk individuals have made it challenging for primary care providers to guide their patients. For instance, the American Cancer Society (ACS) and Centers for Disease Control currently advise starting mammogram screening for average-risk women at age 40, with screenings every one to two years. At the same time, the United States Preventive Services Task Force (USPSTF) suggests initiating screening at age 50, followed by biennial screenings [12]. Such discrepancies pose significant challenges, especially in our academic institution.

Furthermore, the average age at which breast cancer is diagnosed varies by geographical location. In the United States, the average age of diagnosis is 62 years, with only 9% of patients receiving a diagnosis at age 45 or younger [13]. However, in our study, which focused on patients under 50 years, we identified only two cases of breast cancer (n=2, 1.33% of the sample), as in Table 1, occurring at ages 47 and 49. Additionally, disparities exist based on race, with Black American women typically diagnosed at age 60 compared to 62 for White women and a 20% lower incidence rate in the Hispanic population [13]. According to current literature, the breast cancer incidence rate appears to be highest in white non-Hispanic women [13]. And the mortality rate appears to be highest among Black women [14, 15]. In our study, the only cases of breast cancer diagnosed in patients under 50 were one Black American at 49 years and a Hispanic female at 47 years. Notably, most of our study sample consisted of Black individuals (n=100, 66.7%), and 10.7% (n=16) were Hispanic. 

The issue of overdiagnosis in early screening of average-risk women is another concern. This often leads to unnecessary testing and treatment of cancers that may not have posed a threat in a patient's lifetime [16-20]. Moreover, it exposes individuals to physical (radiation, testing costs) and emotional (due to false positives) complications. Current estimates suggest that overdiagnosis from screening mammograms can range from 0% to 30% [21]. The risk of false-positive results is also significant, leading to additional, expensive, invasive, time-consuming, and emotionally challenging tests. In our study, 46 patients (30.7%) underwent further testing (Table 2), including 6.7% (n=10) who required biopsies (Table 3), highlighting the delicate balance between risks and benefits.

Considering the presence or absence of risk factors is crucial. Although our study excluded genetically and environmentally related risk factors, cosmetic motivations, and screenings secondary to physical examinations, other factors like smoking history and race need close consideration when determining the starting age for mammogram screening due to the higher incidence of breast cancer in these populations. In our study, both cases of breast cancer occurred in African and Hispanic populations, although the incidence is generally higher in the White population [13]. Notably, both patients were smokers, consistent with existing literature on a significantly higher risk of breast cancer among women who are current smokers or started smoking at an early age [22]. It's essential to acknowledge that the age of diagnosis in our patients was significantly lower than the average for their respective races (60 years versus 49 years). However, it's vital to recognize that our clinic serves more Black patients (n=100, 66.7%) than White patients (n=26, 17.3%), which could affect detection rates. Our study's sample size is a limitation, and a larger study is recommended to establish a stronger correlation between these factors and the early detection of breast cancer through mammogram screening.

Given that our facility's patient population has at least one risk factor for breast cancer, we recommend continuing early screening at age 40 and continuing annually. Compliance with follow-up remains an issue, as noted in Table 4. However, this should be accompanied by extensive discussions with patients about the risks and benefits of early breast cancer screening. The ultimate decision should be made collaboratively by the provider and patient, with room for adjustments pending the completion of further studies.

Limitations

Although the overall goal of this study is to serve as a quality improvement project that guides mammogram screening in our facility, this study has limitations. The study is based on data from only 150 women, which may not represent the broader population. The study was conducted at a single hospital in Nashville, limiting the generalizability of findings. The participant population is skewed, with a higher proportion of Black individuals and a lower proportion of White individuals, however consistent with our patient population. Some data, especially regarding risk factors and patient histories, were incomplete, which could affect the result of the study. The study's data only goes up to January 2023, potentially missing long-term trends/ findings. The study doesn't compare different screening initiation ages, making it hard to draw clear conclusions. It focuses only on women who had mammogram screenings, excluding those who did not.

The above limitations suggest the need for larger-scale research to establish stronger correlations.

## Conclusions

In conclusion, our study underscores the complexities surrounding mammogram screening for average-risk women aged 40-49. Despite the observed challenges of overdiagnosis and false positives, our findings, based on a limited sample, indicate that early screening may detect breast cancer cases in this age group, particularly among populations with distinct risk factors, such as smoking history. However, the inconsistency in screening guidelines and the impact of demographic factors like race warrant continued discussion between healthcare providers and patients. While our recommendation leans toward early screening at age 40, we emphasize the importance of shared decision-making, with an awareness of the potential benefits and drawbacks of early mammogram screening.

However, it's crucial to acknowledge the limitations of our single-center study with a relatively small sample size, which necessitates further research to establish more robust correlations. Future studies should aim for larger, more diverse populations and longitudinal data to provide a more comprehensive understanding of breast cancer screening practices and outcomes.
